# Superovulation Using the Combined Administration of Inhibin Antiserum and Equine Chorionic Gonadotropin Increases the Number of Ovulated Oocytes in C57BL/6 Female Mice

**DOI:** 10.1371/journal.pone.0128330

**Published:** 2015-05-29

**Authors:** Toru Takeo, Naomi Nakagata

**Affiliations:** Division of Reproductive Engineering, Center for Animal Resources and Development (CARD), Kumamoto University, 2-2-1 Honjo, Chuo-ku, Kumamoto, 860–0811, Japan; University of Missouri, UNITED STATES

## Abstract

Superovulation is a reproductive technique generally used to produce genetically engineered mice. Superovulation in mice involves the administration of equine chorionic gonadotropin (eCG) to promote follicle growth and then that of human chorionic gonadotropin (hCG) to induce ovulation. Previously, some published studies reported that inhibin antiserum (IAS) increased the number of ovulated oocytes in ddY and wild-derived strains of mice. However, the effect of IAS on the C57BL/6 strain, which is the most widely used inbred strain for the production of genetically engineered mice, has not been investigated. In addition, the combined effect of IAS and eCG (IASe) on the number of ovulated oocytes in superovulation treatment has not been examined. In this study, we examined the effect of IAS and eCG on the number of ovulated oocytes in immature female mice of the C57BL/6 strain in superovulation treatment. Furthermore, we evaluated the quality of obtained oocytes produced by superovulation using IASe by *in vitro* fertilization (IVF) with sperm from C57BL/6 or genetically engineered mice. The developmental ability of fresh or cryopreserved embryos was examined by embryo transfer. The administration of IAS or eCG had a similar effect on the number of ovulated oocytes in C57BL/6 female mice. The number of ovulated oocytes increased to about 3-fold by the administration of IASe than by the administration of IAS or eCG alone. Oocytes derived from superovulation using IASe normally developed into 2-cell embryos by IVF using sperm from C57BL/6 mice. Fresh or cryopreserved 2-cell embryos produced by IVF between oocytes of C57BL/6 mice and sperm from genetically engineered mice normally developed into live pups following embryo transfer. In summary, a novel technique of superovulation using IASe is extremely useful for producing a great number of oocytes and offspring from genetically engineered mice.

## Introduction

The use of experimental animals in prospective studies is essential to deeply understand healthy or pathological conditions and to evaluate the efficacy of candidate therapies before being applied to human patients. Mice are the most commonly used experimental animals in life science research. Recently, many lines of genetically engineered mice have been produced to decipher gene functions in biological process or disease model.

A great number of genetically engineered mice have been archived in mouse resource banks and are available from the banks *via* the website of International Mouse Strain Resource [1]. In the mouse resource bank, various reproductive techniques are used to efficiently preserve, transport, or produce genetically engineered mice [2]. Until now, a robust and efficient system for managing the mouse resource bank has been established using cryopreservation techniques for sperm, oocytes, and embryos [3–6]. Cryopreserved samples can be efficiently preserved and easily transported by courier service. In addition, novel techniques have been developed for simply shipping unfrozen mouse embryos or sperm at 4°C; hence, the use of dryshippers and special skills for handling cryopreserved samples is not required [7–10]. Furthermore, a pharmaceutically assisted *in vitro* fertilization (IVF) system has been developed using methyl-β-cyclodextrin and reduced glutathione [11, 12]. This IVF system achieves stable and high rates of fertilization using cryopreserved and cold-stored mouse sperm. Improvement of reproductive techniques is very important to efficiently conduct research using genetically engineered mice.

In this study, we focused on superovulation; it is an important reproductive technique that enables artificial increases in the number of ovulated oocytes. The phenomenon of superovulation involves the induction of follicle maturation and ovulation by hormone administration. The technique is routinely performed to obtain oocytes from oocyte donors before IVF for producing genetically engineered mice. In general, female mice are intraperitoneally injected with equine chorionic gonadotropin (eCG) to stimulate follicle growth and are subsequently injected with human chorionic gonadotropin (hCG) to induce ovulation [13]. The number of ovulated oocytes in the C57BL/6 strain, which is commonly used as the background strain for genetically engineered mice, is approximately 25 per female by following superovulation treatment [14]. Increasing the number of ovulated oocytes by superovulation treatment will clearly reduce the number of oocyte donors and increase the efficiency of animal production. It is important to minimize the number of animals based on the 3Rs principle (reduction, refinement, and replacement) in animal experiments [15]. Therefore, development of a novel technique of superovulation to increase the number of ovulated oocytes is strongly demanded.

Several studies have reported that the administration of inhibin antiserum (IAS) increased the number of ovulated oocytes in various animals such as hamsters, rats, guinea pigs, cows, and mares [16–20]. Inhibin is known to be a hormone secreted by granulosa cells in the ovarian follicle [21], and inhibin secreted into the general circulation acts on the anterior pituitary gland to prevent secretion of follicle stimulating hormone (FSH) from this gland. The combined regulation of the level of inhibin and FSH critically control the timing of follicle growth [22]. The administration of IAS to female rats neutralizes the function of inhibin, resulting in negation of the negative feedback by inhibin against FSH and promotion of follicle growth and increases in the number of ovulated oocytes [20].

Previously, IAS successfully increased the number of ovulated oocytes in female mice of the ddY strain [23, 24]. The effect of IAS was also reported in female mice of wild-derived strains [25, 26]. However, the effect of IAS on the most widely used inbred strain of C57BL/6 mice has not been reported. In addition, the combined effect of IAS and eCG on the number of ovulated oocytes in superovulation treatment has not been examined. In this study, we investigated the effect of IAS and eCG (IASe) on the number of ovulated oocytes in C57BL/6 female mice. In addition, oocytes produced by superovulation using IASe were used for IVF with sperm derived from C57BL/6 or genetically engineered mice. Subsequently, the developmental ability of the fresh or cryopreserved embryos of genetically engineered mice was examined by embryo transfer.

## Materials and Methods

### Animals

Female or male mice of the C57BL/6J strain (purchased from CLEA Japan) were used as oocyte donors at 4 weeks of age or sperm donors at 12 weeks of age. Three strains of genetically engineered mice with a C57BL/6 background were similarly used as sperm donors at 12 weeks of age. ICR mice were used as recipients of 2-cell embryos at 8–16 weeks of age. All animals were housed under a 12-h dark–light cycle (light from 07:00 to 19:00) at 22 ± 1°C with *ad libitum* food and water. The Animal Care and Use Committee of Kumamoto University School of Medicine approved the protocols for animal experiments.

### Media

Sperm were preincubated in a modified Krebs–Ringer bicarbonate solution (TYH) containing 1.0 mg/mL polyvinyl alcohol and 0.75 mM methyl-β-cyclodextrin (Sigma-Aldrich) [11]. Calcium-enhanced human tubal fluid was used as fertilization medium [27, 28]. Potassium simplex optimized medium was used to handle and culture 2-cell embryos to the blastocyst stage [29]. Embryo cryopreservation was performed using 1.0 M dimethyl sulfoxide (DMSO) diluted in modified phosphate-buffered saline (PB1) and 2.0 M DMSO, 1.0 M acetamide, and 3.0 M propylene glycol in diluted PB1 (DAP213) [6]. Vitrified embryos were warmed in PB1 containing 0.25 M sucrose.

### Superovulation and oocyte collection

Female mice were administered IAS (0.1 or 0.2 mL) alone, eCG (3.75 or 7.5 IU, ASKA Pharmaceutical Co. Ltd.) alone, or combined IAS (0.1 mL) and eCG (3.75 IU). IAS was prepared by the method described by Kishi et al. [30]. The titer of IAS was confirmed by ELISA against inhibin peptide and measured the concentration of inhibin antibody after the purification of affinity chromatography (2.0 mg/mL antibody). The dose of 7.5 IU eCG was used as control based on the protocol of superovulation in our previous works [4, 5, 7–12]. The efficacy of IASe (0.1 mL IAS and 3.75 IU eCG) was equal to the double dose of IASe (0.2 mL IAS and 7.5 IU eCG) in our preliminary study. Therefore, we chose the lower dose of IASe to investigate the combined effects of IAS and eCG in this study.

At 48 h after the administration of these reagents, 7.5 IU hCG (ASKA Pharmaceutical Co. Ltd.) was administered to mice. At 17 h after hCG injection, mice were sacrificed by cervical dislocation, and their oviducts were quickly collected and transferred to a fertilization dish covered with paraffin oil. Under microscopic observation, cumulus–oocytes complexes were collected from the oviducts and transferred to a 200-μL drop of fertilization medium. The number of ovulated oocytes and fertilization ability of oocytes in each group were examined.

### IVF

Sperm were collected from C57BL/6 or genetically engineered mice with a C57BL/6 background as follows. Male mice were sacrificed by cervical dislocation, and the cauda epididymides were collected and transferred to a dish of sperm preincubation medium covered with paraffin oil. Clots of sperm were collected from the cauda epididymides using a dissecting needle and transferred to a 100-μL drop of sperm preincubation medium. Sperm were preincubated for 60 min to induce capacitation and then added to the fertilization drop containing cumulus–oocytes complexes and cultured with oocytes at 37°C in an atmosphere containing 5% CO_2_ for 3 h. The final concentration of motile sperm in fertilization medium was 400–800 sperm/μL. At 3 h after insemination, oocytes were washed in 3 drops of human tubal fluid (80 μL) and the number of ovulated oocytes was examined. At 24 h after insemination, fertilization rates were calculated as the total number of 2-cell embryos divided by the total number of inseminated oocytes multiplied by 100.

### Embryo vitrification and warming

Embryo vitrification was performed according to described previously procedures [6]. After IVF, 2-cell embryos of C57BL/6 or genetically engineered mice were introduced into about a 100-μL drop of the DMSO solution and then transferred into other drops of the DMSO solution. An aliquot of 5 μL the DMSO solution containing the embryos was placed and equilibrated in a cryotube for 5 min at 0°C. An aliquot of 45 μL DAP213 was added to the cryotube and incubated for 5 min at 0°C. After the equilibration for 5 min, the cryotube containing the embryos was then immersed and preserved in liquid nitrogen. The vitrified embryos in the cryotube were warmed by adding 0.9 mL sucrose solution pre-warmed to 37°C. The vitrified-warmed embryos were collected from the sucrose solution and then transferred into a drop of 100 μL potassium simplex optimized medium. After 10 min, survival rate was examined by counting the number of morphologically normal embryos before embryo transfer.

### Embryo transfer

Embryo transfer was performed as previously described [31]. Freshly harvested or vitrified-warmed 2-cell embryos of the C57BL/6 or genetically engineered mice were transferred into the oviducts of ICR females (7–11 embryos/oviduct) on the day a vaginal plug was present (Day 1 of pseudopregnancy). The embryos were transferred through the wall of the fallopian tube. The number of offspring was recorded after 19 days.

### Statistics

Statistical analysis was performed using Prism version 5.0 (GraphPad). Results are expressed as the mean ± standard deviation (SD). Group results were compared using analysis of variance after arcsine transformation of the percentages; *P* < 0.05 was considered statistically significant.

## Results

### Combined administration of IAS and eCG increased the number of ovulated oocytes in C57BL/6 female mice

Three types of administration (IAS alone, eCG alone, and IASe) were examined for their ability to induce superovulation. The administration of IASe (0.1 mL IAS + 3.75IU eCG) was the most effective to increase the number of ovulated oocytes compared with IAS or eCG alone (Table 1). The number of ovulated oocytes induced by the administration of the IASe was about 3-fold higher than the number induced by the administration of 0.2 mL IAS or 7.5 IU eCG. The effect of 0.1 or 0.2 mL IAS was equal to that of 7.5 IU eCG. Fertilization ability of oocytes was not different in all groups.

**Table 1:** Effect of IAS, eCG, or IAS + eCG on the number of ovulated oocytes and fertilization rate of C57BL/6 mice.

IAS (mL)	eCG (IU)	Total no. of ovulated oocytes	Average no. of oocytes/female	No. of 2-cell embryos	Fertilization rate (%)
0.1	0	321	32.1 ± 16.6^†^	310	96.6 ± 3.0
0.2	0	365	36.5 ± 12.5^†^	342	93.7 ± 4.7
0	3.75	87	8.7 ± 1.7^*^ ^,^ ^**^	84	96.6 ± 15.8
0	7.5	277	27.7± 5.4^†^	267	96.4 ± 3.2
0.1	3.75	1072	107.2 ± 22.7^*^ ^,^ ^**^ ^,^ ^†^ ^,^ ^‡^	963	89.8 ± 3.7

Ten female mice were used as oocyte donors in each group. The results are expressed as mean ± SD.

^*^ *P* < 0.05 compared with 0.1 mL IAS

^**^ *P* < 0.05 compared with 0.2 mL IAS

^†^ *P* < 0.05 compared with 3.75 mL eCG

^‡^ *P* < 0.05 compared with 7.5 mL eCG.

### Many 2-cell embryos of genetically engineered mice were produced from superovulated oocytes obtained by IASe treatment

To evaluate the efficiency of embryo production of genetically engineered mice using the oocytes derived from the IASe-treated female mice, IVF was performed using 3 strains of genetically engineered mice as sperm donors. The efficiency of embryo production was compared with the IVF using oocytes derived from eCG treatment in C57BL/6 mice as control. IASe increased the number of ovulated oocytes compared with eCG (Table 2). More than 100 oocytes were obtained from a female mouse by the IASe treatment. The fertilization rate of IVF between the oocytes by IASe treatment and sperm of genetically engineered mice was slightly lower than that of IVF using oocytes by eCG treatment and sperm in C57BL/6 mice. The embryos were transferred to recipients before or after cryopreservation in a following experiment.

**Table 2:** Production of 2-cell embryos by IVF between superovulated oocytes of C57BL/6 mice obtained by IASe treatment and sperm of genetically engineered mice.

	Oocyte donor		No. of	No. of	
Treatment	ID	Sperm donor	ovulated oocytes	2-cell embryos	Fertilization rate (%)
eCG	1	C57BL/6	35	35	100.0
	2	C57BL/6	36	36	100.0
	3	C57BL/6	30	30	100.0
	4	C57BL/6	38	37	97.4
	5	C57BL/6	36	35	97.2
	6	C57BL/6	42	41	97.6
		Average	36.2±3.9	35.7±3.6	98.6±1.4
IASe	7	Strain A	107	101	94.4
	8	Strain A	115	112	97.4
	9	Strain B	110	106	96.4
	10	Strain B	110	104	94.5
	11	Strain C	111	99	89.2
	12	Strain C	100	86	86.0
		Average	108.8±5.0*	101.3±8.8*	93.1±4.9*

Superovulation was induced by 7.5 IU eCG or IASe (0.1 mL IAS and 3.75 IU eCG). The eCG (oocyte donor ID: 1–6) or IASe (oocyte donor ID: 7–12) was administered to 6 female mice in each group. IVF was performed between oocytes of the C57BL/6 mouse strain and sperm of C57BL/6 mouse or from genetically engineered mice. Three strains of genetically engineered mice (Strains A, B, or C) were used as sperm donors. The results are expressed as mean ± SD.

^*^ *P* < 0.05 compared with eCG.

### Offspring were obtained from the fresh or cryopreserved embryos derived from superovulation using IASe

The developmental ability of the fresh or cryopreserved 2-cell embryos derived from the IASe treatment was examined by embryo transfer. After embryo transfer, all recipients delivered live pups derived from the superovulated oocytes obtained by IASe treatment (Table 3). The birth rates were not different between eCG and IASe in the groups of fresh or cryopreserved embryos. The average number of offspring derived from a female mouse superovulated by IASe was 2 to 3 times higher than that of eCG.

**Table 3:** Development rate of the fresh and cryopreserved 2-cell embryos derived from IVF using superovulated oocytes obtained by IASe treatment.

Embryos	Treatment	Oocyte donor ID	No. of cryopreserved embryos	No. of recovered embryos	No. of survived embryos	No. of recipients	No. of transferred embryos	No. of offspring	Birth rate (%)
		1	-	-	-	2	35	18	51.4
	eCG	2	-	-	-	2	36	15	41.7
		3	-	-	-	2	30	11	36.7
Fresh		Average	-	-	-	2	33.7±3.2	14.7±3.5	43.6±7.5
		7	-	-	-	5	101	59	58.4
	IASe	9	-	-	-	5	106	48	45.3
		11	-	-	-	5	99	41	41.4
		Average	-	-	-	5	102±3.6*	49.3±9.1*	48.4±8.9
		4	37	37	32	2	32	17	53.1
	eCG	5	35	33	27	2	27	16	59.3
		6	41	41	39	2	39	12	30.8
Cryopreserved		Average	37.7±3.1	37.0±4.0	32.7±6.0	2	32.7±6.0	15±2.6	45.9±15.0
		8	112	111	100	5	100	51	51
	IASe	10	104	101	84	5	84	32	38.1
		12	86	85	85	5	85	35	41.2
		Average	100.7±13.3*	99.0±13.1*	89.7±9.0*	5	89.7±9.0*	39.3±10.2*	43.9±10.2

After IVF between oocytes of C57BL/6 mice and sperm from genetically engineered mice, the 2-cell embryos without (Oocyte donor ID: eCG; 1–3 and IASe; 7, 9, 11) or with cryopreservation (Oocyte donor ID: eCG; 4–6 and IASe; 8, 10, 12) were used for embryo transfer. The results are expressed as mean ± SD.

^*^ *P* < 0.05 compared with eCG in each group of the fresh or cryopreserved embryos.

## Discussion

**Fig 1:**
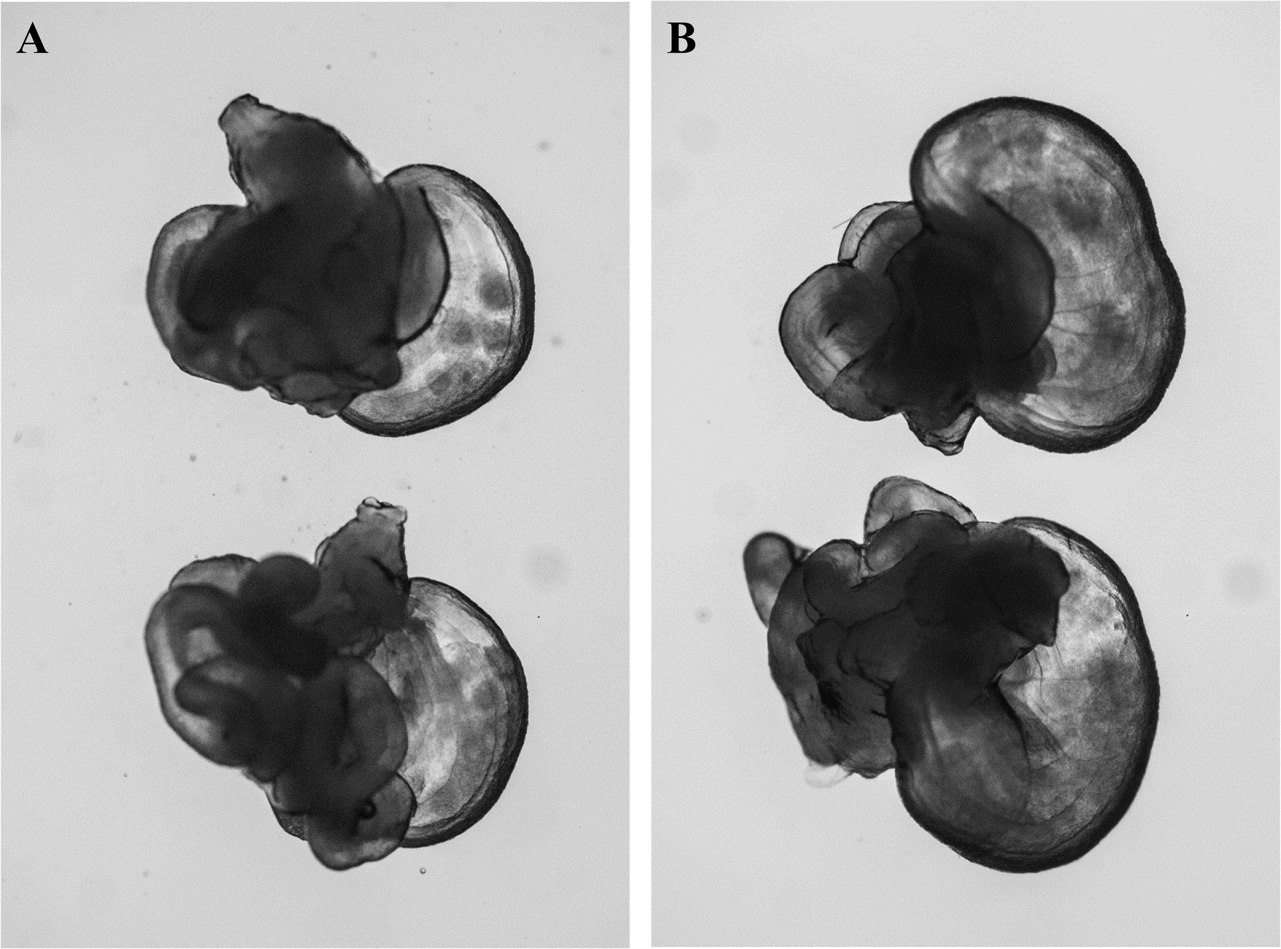
Observation of the swollen ampulla of oviducts after injection of 7.5 IU eCG (A) or IASe (0.1 mL IAS and 3.75 IU eCG) (B).

**Fig 2:**
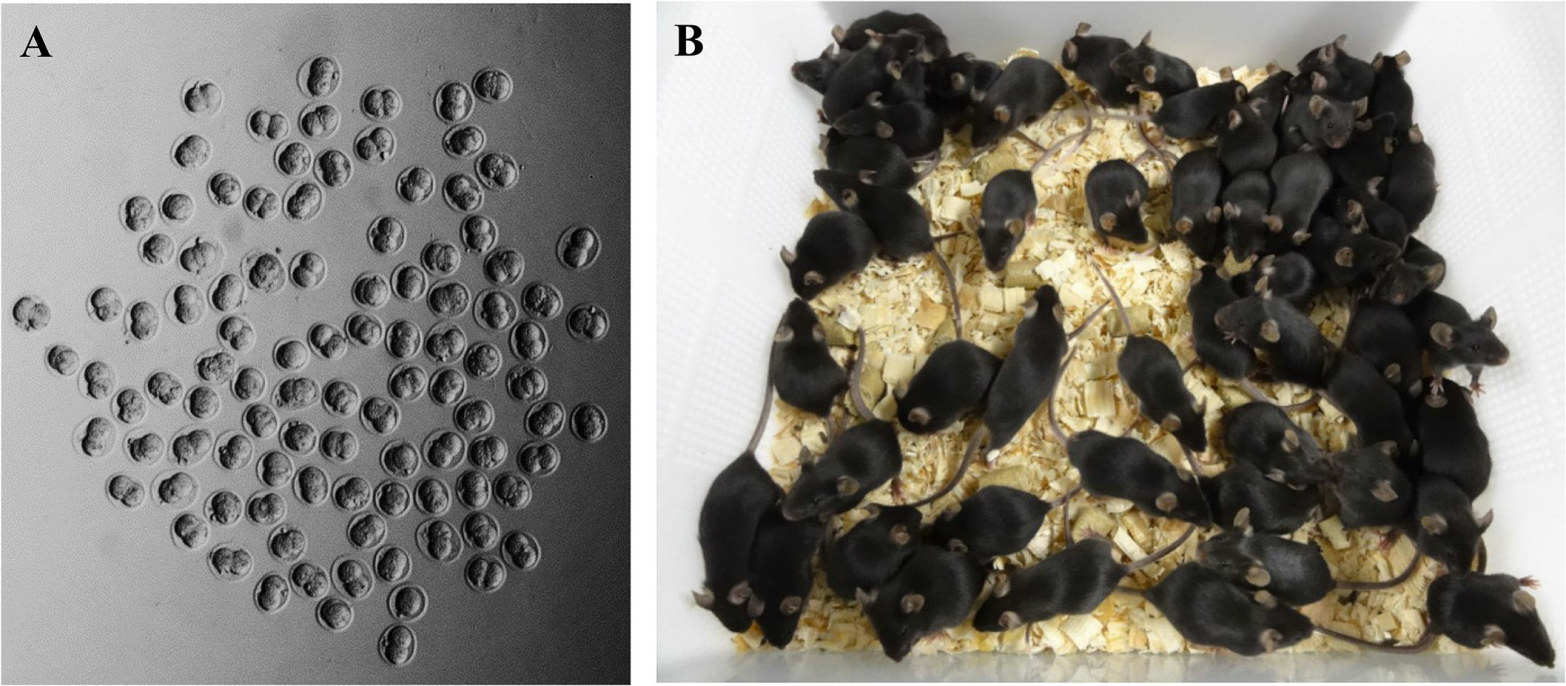
Production of 2-cell embryos (A) and live pups (B) from single female mice superovulated using IASe (0.1 mL IAS and 3.75 IU eCG).

In the present study, we demonstrated that the combined administration of IAS and eCG greatly increased the number of ovulated oocytes in C57BL/6 female mice compared with the conventional treatment of superovulation using eCG or IAS alone. The ovulated oocytes by treatment with IASe had fertilizing ability but the fertilization rate of IASe slightly declined. After the embryo transfer, normal offspring was obtained from fresh or cryopreserved 2-cell embryos produced by IVF between oocytes of C57BL/6 female mice treated with IASe and sperm from genetically engineered mice. This is the first study to stably produce more than 80 oocytes and 30 offspring from a single female C57BL/6 mouse using the novel method of superovulation using IASe (Tables 2 and 3, Figs 1 and 2).

Superovulation is an important technique to efficiently produce and obtain oocytes from oocyte donors in animal experiments. In superovulation in mice, eCG is intraperitoneally administered to female mice; at 48 h after eCG administration, hCG is subsequently administered to mice in the same manner, and then, oocytes are collected at 14–17 h after hCG administration [13]. The above technique has been widely accepted as the standard protocol for superovulation since it was established. In this study, we demonstrated the administration of optimal dose of IAS (0.1 or 0.2 mL) increased the number of ovulated oocytes to a number that was as same as that obtained with the administration of 7.5 IU eCG. Lower (0.05 mL) or higher (0.4 mL) doses of IAS also indicated the ability to induce superovulation. However, the number of ovulated oocytes was unstable in the range of IAS (data not shown). Interestingly, we revealed that the number of ovulated oocytes was dramatically enhanced by the combined administration of IAS and eCG in C57BL/6 female mice. This improved superovulation technique can surely provide efficient production of oocytes and animals to enhance the efficiency of experiments using genetically engineered mice.

Immunoneutralization of endogenous inhibin by IAS is an efficient strategy for inducing superovulation in mice. Wang et al. first reported that the administration of 200 μL IAS, instead of eCG, increased the number of ovulated oocytes in immature and adult ddY mice [23]. Medan et al. showed IAS-treated female ddY mice had a significantly increased concentration of plasma FSH [24]. FSH elevation likely contributed to the promotion of follicle growth and increased the number of ovulated oocytes. Hasegawa et al. similarly reported 50 μL of IAS increased the number of oocytes in Japanese wild-derived strains belonging to *Mus musculus molossinus* (MSM/Ms and JF1/Ms), which are poor responders to eCG [25]. The best age for the administration of IAS to female mice was 5–7 weeks in MSM/Ms and JF1/Ms. Recently, Mochida et al. compared the effect of eCG or IAS on the number of ovulated oocytes in various wild-derived strains from 5 subspecies of *Mus musculus* [26]. The responsiveness to eCG or IAS was strongly dependent on their genetic background. They demonstrated female mice belonging to *Mus musculus molossinus* tended to be highly responsive to IAS compared with eCG. On the other hand, *Mus musculus domesticus* showed low responsiveness to IAS compared with eCG. The C57BL/6 strain is known to belong to *Mus musculus domesticus* [32, 33]. In this study, we demonstrated that the responsiveness to IAS (0.1 or 0.2 mL) or eCG (7.5 IU) administered for superovulation in female mice of the C57BL/6 strain was similar (Table 1) and that this responsiveness was enhanced by the combined administration of IAS and eCG. The novel administration of IASe may also be effective in increasing the number of ovulated oocytes in wild-derived strains regardless of the genetic background. However, further experiments need to be conducted to examine the application of superovulation using IASe to various subspecies of *Mus musculus* and to elucidate their combined effect on elevating the number of ovulated oocytes.

Now, numerous commercial reagents of eCG are available via pharmaceutical or biotechnological companies. On the other hand, there is no commercial reagent of the IAS. Therefore, IAS need to be prepared by ourselves and evaluated the titer, effectiveness and sterile conditions of the reagents before use. In the future, the accessibility of the IAS should be improved to prevail the superovulation technique using IASe in scientific community.

In conclusion, we developed a novel technique of superovulation using the administration of IASe to C57BL/6 mice. The technique can greatly increase the number of ovulated oocytes compared with conventional methods using eCG. Our newly developed technique can minimize the number of female mice required as oocyte donors, which meets the principle of 3Rs for good laboratory animal practice conduct. We strongly believe that enhancement of the efficiency of animal production using this superovulation technique will improve the activity of mouse resource banks and transgenic labs, which can lead to significant progress in genome research using genetically engineered mice.
